# Characterization of SNF472 pharmacokinetics and efficacy in uremic and non-uremic rats models of cardiovascular calcification

**DOI:** 10.1371/journal.pone.0197061

**Published:** 2018-05-09

**Authors:** Miguel D. Ferrer, Markus Ketteler, Fernando Tur, Eva Tur, Bernat Isern, Carolina Salcedo, Pieter H. Joubert, Geert J. Behets, Ellen Neven, Patrick C. D’Haese, Joan Perelló

**Affiliations:** 1 Laboratoris Sanifit SL, Research and Development Department, Palma, Spain; 2 Department of Fundamental Biology and Health Sciences, University of the Balearic Islands, Palma, Spain; 3 Division of Nephrology, Klinikum Coburg GmbH, Coburg, Germany; 4 University Institute of Health Sciences Research, University of the Balearic Islands, Palma, Spain; 5 Institute of Pharmaceutical Science, King’s College, London, United Kingdom; 6 Laboratory of Pathophysiology, Department of Biomedical Sciences, University of Antwerp, Antwerp, Belgium; University Medical Center Utrecht, NETHERLANDS

## Abstract

End-stage renal disease is strongly associated with progressive cardiovascular calcification (CVC) and there is currently no therapy targeted to treat CVC. SNF472 is an experimental formulation under development for treatment of soft tissue calcification. We have investigated the pharmacokinetics of SNF472 administration in rats and its inhibitory effects on CVC. SNF472 was studied in three rat models: (1) prevention of vitamin D_3_-induced CVC with an intravenous SNF472 bolus of 1 mg/kg SNF472, (2) inhibition of progression of vitamin D_3_-induced CVC with a subcutaneous SNF472 bolus of 10 or 60 mg/kg SNF472, starting after calcification induction, (3) CVC in adenine-induced uremic rats treated with 50 mg/kg SNF472 via i.v. 4h -infusion. Uremic rats presented lower plasma levels of SNF472 than control animals after i.v. infusion. CVC in non-uremic rats was inhibited by 60–70% after treatment with SNF472 and progression of cardiac calcification completely blocked. Development of CVC in uremic rats was inhibited by up to 80% following i.v. infusion of SNF472. SNF472 inhibits the development and progression of CVC in uremic and non-uremic rats in the same range of SNF472 plasma levels but using in each case the required dose to obtain those levels. These results collectively support the development of SNF472 as a novel therapeutic option for treatment of CVC in humans.

## Introduction

Advanced chronic kidney disease (CKD) and end-stage renal disease (ESRD) are strongly associated with progressive cardiovascular calcification (CVC)[1–4]. Disturbances in calcium (Ca) and phosphorus (P) metabolism are among the key elements that trigger extraosseous calcification in CKD. Calcification of the vessel wall is a highly regulated active process involving transdifferentiation of vascular smooth muscle cells (VSMC) into an osteoblast-like phenotype[5, 6]. This process is counteracted by circulating or local inhibitors of calcification, such as pyrophosphate, fetuin-A, osteopontin or matrix-Gla protein. A decrease in serum or tissue levels of these inhibitors may contribute to CVC and subsequent cardiovascular disease in CKD[7].

Strategies to control parathyroid hormone (PTH), Ca^2+^ and P levels (including dietary manipulation, managing vitamin D status and drug therapy) are currently applied for management of advanced CKD. The key medical treatments to date are vitamin D analogs to increase plasma vitamin D levels, phosphate binders to reduce hyperphosphatemia, and calcimimetics to control PTH secretion[8, 9]. Results from the ADVANCE study suggested, but did not demonstrate conclusively, that cinacalcet attenuates vascular and cardiac valve calcification progression in patients on hemodialysis (HD)[10]. The use of non-calcium containing phosphate binders, such as sevelamer, has additionally been associated with slower progression of cardiovascular calcification and a significant survival benefit, compared to calcium-containing phosphate binders that can induce hypercalcemia and therefore enhance cardiovascular calcification per se [11–13].

SNF472, the hexasodium salt of myo-inositol hexaphosphate (IP6, phytate), is a potent calcification inhibitor. It inhibits the development and progression of ectopic calcifications by binding to the growth sites of the hydroxyapatite (HAP) crystal, the main component of CVC deposits. This effect appears to be independent of the etiology of CVC and is present at any plasma calcium (Ca) and/or phosphate levels [14].

Thus, phytate (the active ingredient of SNF472) is a naturally occurring substance found in beans, rice, corn and other high-fiber foods that is also present in mammalian cells and tissues at micromolar concentrations [15]. Following detection of significant levels in human urine [16], a link between phytate and human health was established, particularly in the context of diseases related to disruption of calcium levels. In this context, consumption of phytate in humans or treatment with phytate in animal models has been related to positive effects against pathological conditions such as renal calculi [17–19], osteoporosis [20–22] and cardiovascular calcification [23–25].

Previous studies on inhibition of CVC by phytate in animal models focused on oral[26] or topical[23] administration, but the effects of i.v. phytate on CVC in a uremic animal model are yet to be investigated. In addition, none of these previous studies on the effects of phytate administration in calcium-related pathologies have reported its circulating levels, thus avoiding establishing a clear relation between the levels of phytate in blood and its effect on CVC. Therefore, our primary aim was to study the pharmacokinetics (PK) of SNF472 after single subcutaneous (s.c.), i.v. bolus and i.v. infusion administration to rats and explore the effects of the drug on CVC in three different rat models, including uremic rats, to assess its *in vivo* efficacy.

## Materials and methods

### Pharmacokinetics of SNF472

#### Pharmacokinetics of SNF472 administered as an i.v. bolus

Fifty-four Wistar rats weighing ± 315 g were distributed into three groups of 18 (9 males and 9 females per group) receiving different i.v. doses (1, 5, and 10 mg/kg) of SNF472. Blood was collected at 5, 15, 60 and 120 min.

#### Pharmacokinetics of SNF472 administered as a s.c. bolus

Thirty-five male Wistar rats weighing ± 350 g were randomly distributed into three groups receiving 10, 30, or 100 mg/kg SNF472 via s.c. injection. Blood was collected at 0, 5, 10, 15, 20, 25, 30, 60, 120, 180 and 240 min.

#### Pharmacokinetics of SNF472 administered via i.v. infusion in control and uremic rats

The experiment was performed based on a model described by Terai and co-workers[27]. Uremia was induced in 10 male Wistar rats weighing ± 275 g via 10 daily oral administrations of 600 mg/kg adenine (days 1–10). Two further oral doses of 300 ng/kg α-calcidol were administered on days 11 and 13. Animals were divided into two randomized groups receiving either 10 or 50 mg/kg SNF472 daily as a 4 h i.v. infusion. Blood was collected on days 1 (control group) and 14 (uremic group) at 0, 10, 30, 60 and 240 min during the infusion period.

#### Pharmacokinetic parameters

Due to the limitations in obtaining many sequential blood samples from rats, blood samples were only acquired from each animal at three of the nine time-points. Consequently, the area under the curves (AUCs) could not be determined for individual animals and mean AUCs were therefore used to assess dose linearity.

The pharmacokinetic parameters were calculated as indicated in Table 1.

**Table 1:** Pharmacokinetic parameters calculated.

	Definition	Determination
**AUC**_**(0→t)**_ **(ng·h/mL)**	Area Under the Curve from 0 to the time of the last quantifiable concentration	Trapezoidal rule
**AUC**_**(inf)**_ **(ng·h/mL)**	Area Under the Curve from 0 to infinity	${AUC}_{\left(inf\right)}\;=\;{AUC}_{\left(0\to t\right)}+\frac{C_{t}}{\lambda_{z}}$
**AUMC**_**(last)**_ **(ng·h**^**2**^**/mL)**	Area under the first moment curve to the time of the last quantifiable concentration	Trapezoidal rule
**AUMC**_**(inf)**_ **(ng·h**^**2**^**/mL)**	Area Under the first Moment Curve from time 0 to infinity	${AUMC}_{\left(inf\right)}\;=\;{AUMC}_{\left(last\right)}+\frac{C_{t}\;*\;t_{last}}{\lambda_{z}}+\frac{C_{t}}{{\lambda_{z}}^{2}}$
**C**_**0**_ **(ng/mL)**	Initial (or fictive) concentration at time zero for bolus iv adm	
**Cl (L/h/kg)**	Total body clearance (iv adm) (after i.v. adm, F = 1)	$Cl\;=\;\frac{F\;*\;D}{{AUC}_{\left(inf\right)}}$
**λ**_**z**_	Apparent first order terminal elimination rate constant	Estimated terminal slope of the linear regression of log-transformed concentration vs. time curve
**MRT**_**(0→∞)**_ **(h)**	Mean Residence Time	$MRT\;=\;\frac{{AUMC}_{\left(inf\right)}}{{AUC}_{\left(inf\right)}}$
**t**_**1/2**_ **(min)**	Terminal elimination half-life or apparent terminal elimination half-life	$t_{1/2}\;=\;\frac{Ln2}{\lambda_{z}}$
**V**_**z**_ **(L/kg)**	Volume of distribution at terminal phase (iv adm)	$V_{z}\;=\;\frac{Cl}{\lambda_{z}}$

AUC: area under the curve; AUMC: Area Under the first Moment Curve; C_0_: extrapolated concentration at time = 0 min; D: Dose; Cl: clearance; F: bioavailability; **λ**_**z**_: Apparent first order terminal elimination rate constant; MRT: mean residence time; t_1/2_: half-life; Vz: volume of distribution.

#### Phytate (active ingredient of SNF472) quantification by UPLC^®^-MS

Following sampling, blood was collected into K_3_EDTA anticoagulant tubes, centrifuged at 3,500 rpm for 10 min at 4 °C and stored at -80°C pending quantification via UPLC^®^-MS using the method described by Tur et al. [28]

### Inhibitory effect of SNF472 on cardiovascular calcification

#### Prevention of calcification induced by vitamin D

**Fig 1:**
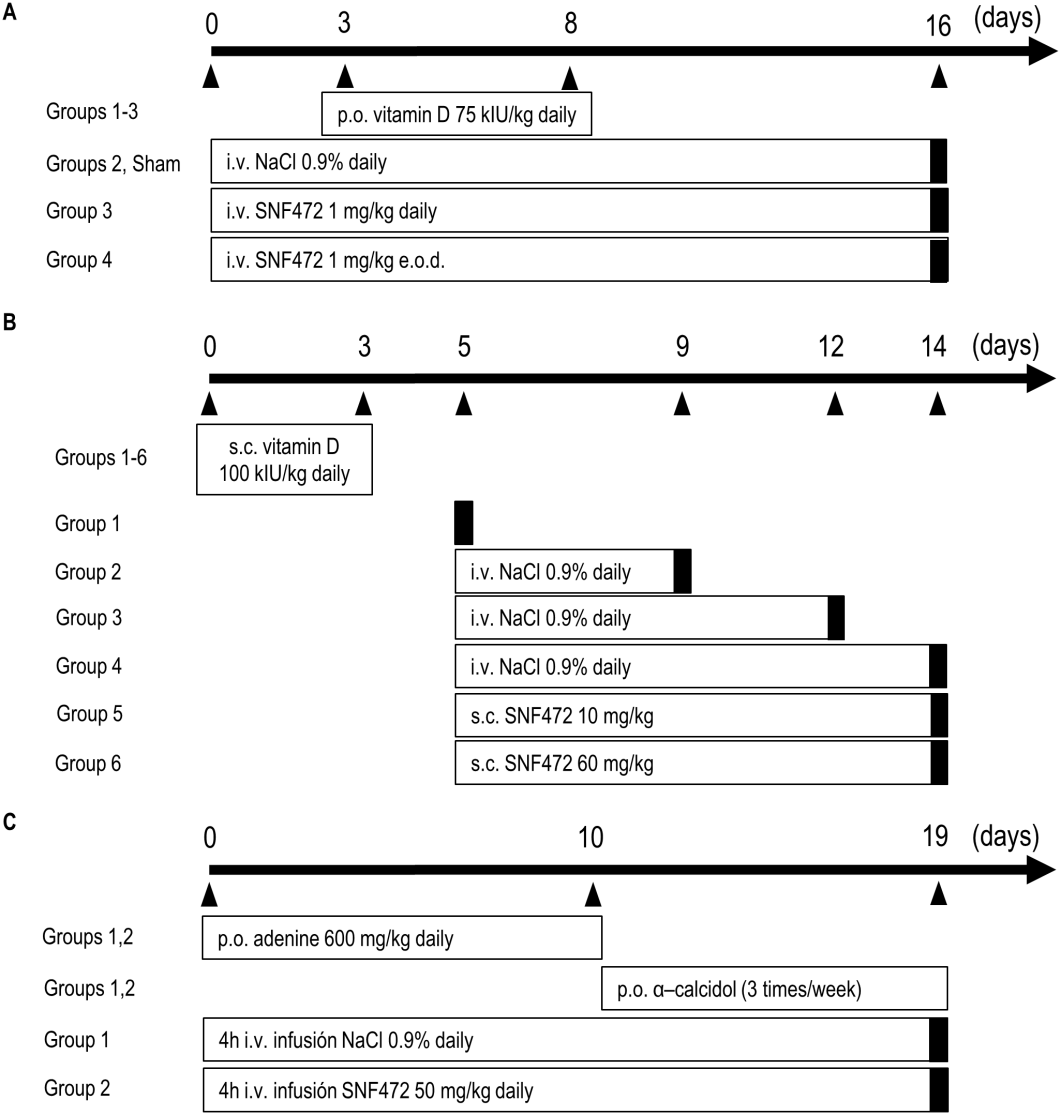
Experimental protocols for *in vivo* analysis of SNF472 efficacy. (A) Prevention of calcification induced by vitamin D. (B) Inhibition of calcification progression induced by vitamin D. (C) Inhibition of calcification in a rat model of uremia. Black bars indicate day of sacrifice. e.o.d., every other day; i.v., intravenous; p.o., peroral; s.c., subcutaneous.

Experiments were performed with 20 male Crl:OF Sprague Dawley rats weighing ± 350 g (Charles River Laboratories International, Inc. L’Arbresle, France). Animals were divided into four groups. The experimental design is summarized in Fig 1a. Group 1 received i.v. vehicle (NaCl 0.9%) daily, Group 2 received i.v. SNF472 (1 mg/kg) daily and Group 3 received i.v. SNF472 (1 mg/kg) every other day (e.o.d.). Calcification was induced by 5 consecutive daily oral administrations of 75 kIU/kg vitamin D_3_ (Fort Dodge, Girona, Spain), starting on day 3 of treatment. Five sham-treated animals with no calcification induction served as controls. Rats were sacrificed 14 days after induction of calcification, and organs collected. Blood was obtained via heart puncture in anesthetized animals immediately before sacrifice.

#### Inhibition of calcification progression induced by vitamin D

Experiments were performed with 48 male Crl:OF Sprague Dawley rats weighing ± 250 g (Charles River Laboratories International, Inc. L’Arbresle, France) divided into 6 groups. Calcification was induced by 3 consecutive daily s.c. administrations of 100 kIU/kg vitamin D_3_ (Fort Dodge, Girona, Spain). Four groups were used as controls to evaluate calcification progression throughout the 14 days of study. The animals received daily s.c. injections of vehicle (NaCl 0.9%), starting 5 days after calcification induction. Groups 5 and 6 received daily s.c. injections of 10 mg/kg SNF472 and 60 mg/kg SNF472, respectively, both starting 5 days after calcification induction. Group 1 animals were sacrificed on day 5 to evaluate calcification. Groups 2 and 3 were sacrificed on days 9 and 12, respectively, to follow the progression of calcification in control animals. Groups 4, 5 and 6 were sacrificed on day 14. Blood was obtained via heart puncture in anesthetized animals immediately before sacrifice. The study design is presented in Fig 1b.

#### Inhibition of calcification in a rat model of uremia

The study was performed with 24 male Crl:WI (Han) Wistar rats weighing ± 250 g (Charles River Laboratories International, Inc. L’Arbresle, France) fed a pelleted high-phosphorus diet (SM R, 10 mm pellets, 1.06% Ca, 1.03% P) (SSNIFF Spezialdiäten, Soest, Germany) ad libitum. Rats were divided into two groups of 12 animals each, according to the scheme presented in Fig 1c. Group 1 received 4 h i.v. infusions of saline (NaCl 0.9%) daily while Group 2 received 4 h i.v. infusions of 50 mg/kg SNF472 daily. All rats were treated daily with adenine (600 mg/kg, suspended in 1% carboxymethyl cellulose, administered orally) for the first 10 days to induce uremia and α-calcidol (300 ng/kg in olive oil, administered orally) three times a week from days 11 to 19 to accelerate and homogenize the development of cardiovascular calcification. On day 19, all surviving animals were sacrificed and blood and tissue samples collected. Blood was obtained via heart puncture in anesthetized animals immediately before sacrifice.

#### Calcium and phosphorus quantification in serum and tissue samples

Aorta and heart were removed at sacrifice, lyophilized for 24 h and weighed. Lyophilized organs were digested using a 1:1 HNO_3_:HClO_4_ mixture in a dry bath incubator for 2–4 h at 180°C and subsequently diluted with MilliQ^™^ water to a final volume of 10 ml. Calcium and phosphorus contents in serum, heart and aorta were quantified via inductively coupled plasma optical emission spectrometry (ICP-OES) (Perkin-Elmer SL, Optima 5300DV spectrometer)[26].

#### Biochemical determinations in uremic rats

Alkaline phosphatase (ALP) activity (ref. OSR6103), and creatinine (OSR6178) and urea (ref. OSR6134) levels were determined in serum using the Beckman Coulter assay kit (Brea, CA, USA).

### In vitro determination of SNF472 binding and release kinetics on hydroxyapatite

To examine SNF472 binding kinetics on hydroxyapatite (HAP), 130 mg of HAP was incubated at 37°C, pH 7.4, with 400 ml of 5,000 ng/ml SNF472. HAP was recovered at different time-points via filtration and dissolved in 0.08% HNO_3_. The solution was transferred to a chromatographic column containing an AG 1-X8 (Bio-Rad, Hercules, CA, USA) anion exchange resin to separate SNF472 from inorganic phosphate, as published previously [29]. SNF472 binding was determined through quantification of total phosphorus in eluate using ICP-OES.

To study SNF472 release kinetics, 400 ml of 5,000 ng/ml SNF472 was initially pre-incubated for 2 h at 37°C with 130 mg HAP. SNF472-preincubated HAP was recovered by filtration and incubated for up to 3 days in fresh 0.05 M Tris buffer, pH 7.40, in the absence of SNF472. HAP was recovered at different time- points by filtration and the total amount of SNF472 attached on the target surface quantified via total phosphorus content determination using ICP-OES after purification via AG 1-X8 anion exchange chromatography, as described previously [29].

### Statistical analysis

Statistical analysis was conducted using a statistical package for social sciences (SPSS for Windows). Results were expressed as means ± SD (except stated otherwise), and p < 0.05 considered statistically significant. The size of the samples was chosen based on previously published data obtained with the same experimental models [25, 27]. Normality of data was assessed with the Shapiro-Wilk test. Only aorta and heart calcium contents in the adenine model were not normally distributed. The statistical significance of data following normal distribution was assessed via Student’s t-test for unpaired data (comparing two groups) or one-way ANOVA (comparing three or more groups) with Fisher’s least significant difference (LSD) post hoc tests when a significant effect was detected using the ANOVA analysis. The statistical significance of non-normally distributed data was assessed with the Mann-Whitney U test. A Kaplan-Meier analysis of survival was also performed in the experiment with uremic animals due to the high mortality observed.

### Study approval

All procedures were performed according to the National Institutes of Health Guide for the Care and Use of Laboratory Animals 85–23 (1996). All the studies in nonuremic animals were approved by the Ethics Committee for Animal Experiments of the University of the Balearic Islands (Palma, Spain). The studies in uremic animals were approved by the Animal Ethics Committee of Charles River (Lyon, France). Animals were killed by carbon dioxide inhalation and exsanguination.

### Materials

Adenine sulfate (Fisher Scientific, Madrid, Spain), α-calcidol (Etalpha, Leo Pharma A/S, Ballerup, Denmark), vitamin D_3_ (Fort Dodge, Girona, Spain), carboxymethyl cellulose (Sigma Aldrich, Madrid, Spain), nitric acid (Sigma Aldrich, Madrid, Spain), perchloric acid (Sigma Aldrich, Madrid, Spain), ICP-OES (Perkin-Elmer SL, Optima 5300DV spectrometer), ALP, creatinine and urea kits (Beckman Coulter, Brea, CA, USA), AG 1-X8 resin (Bio-Rad, Hercules, CA, USA).

## Results

### Pharmacokinetics of SNF472

**Fig 2:**
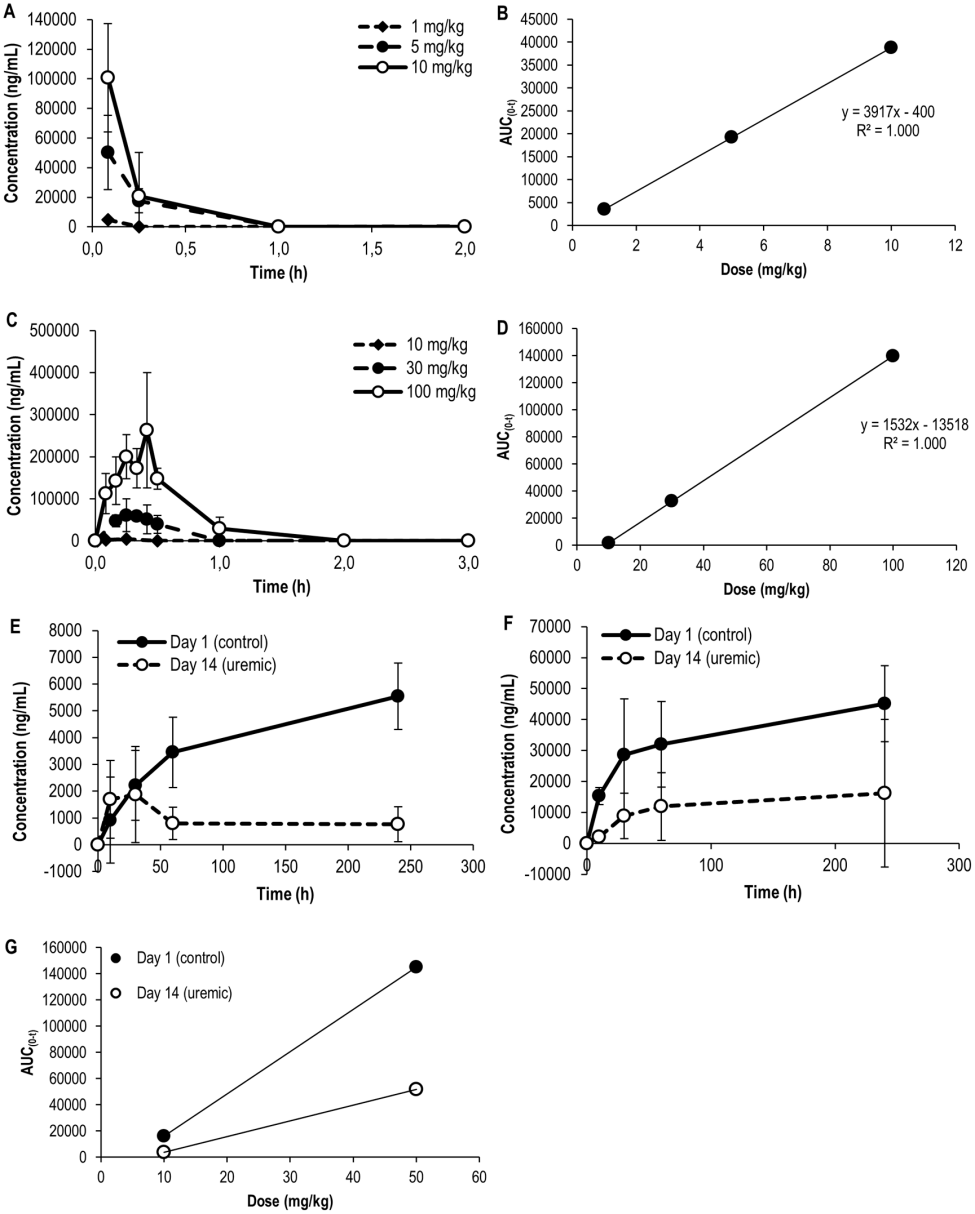
Pharmacokinetics of SNF472 after intravenous (A, B) and subcutaneous (C, D) bolus administration and intravenous infusion into rats (E-G). Phytate levels quantified in plasma samples of control Wistar rats after intravenous (A) and subcutaneous (C) bolus administration (N = 3) and in control and uremic Wistar rats after intravenous infusion of 10 mg/kg (E) and 50 mg/kg SNF472 (F) (N = 5). Results are expressed as means ± SD. Dose-exposure relationship after intravenous (B) and subcutaneous (D) bolus administration and after intravenous infusion (G).

The extent of exposure to phytate (AUC), the active ingredient of SNF472, increased with i.v. doses, indicating a trend towards dose proportionality (Table 2, Fig 2a and 2b, R^2^ = 1.00). However, the levels dropped sharply after i.v. infusion, showing a short half-life (1.8 min for 1 mg/kg and 7–8 min for 5 and 10 mg/kg SNF472).

**Table 2:** Pharmacokinetic parameters after single intravenous bolus administration of SNF472 into rats.

	1 mg/kg	5 mg/kg	10 mg/kg
**C**_**0**_ **(ng/mL)**	36775	84561	156750
**t**_**1/2**_ **(min)**	1.8	6.6	7.8
**AUC**_**(0→t)**_ **(ng·h/mL)**	3491	19234	38752
**MRT**_**(0→∞)**_ **(h)**	1.65	0.33	0.19
**Vz (L/kg)**	0.01	0.04	0.05
**Cl (L/h/kg)**	0.29	0.26	0.26

C_0_: extrapolated concentration at time = 0 min; t_1/2_: half-life; AUC: area under the curve; MRT: mean residence time; Vz: volume of distribution; Cl: clearance

SNF472 was absorbed when administered subcutaneously (Fig 2c), with t_max_ between 15 and 25 min. Exposure to phytate after s.c. administration also showed AUC dose proportionality (Fig 2d, R^2^ = 1,00) with a similar half-life range to that observed after i.v. administration (4–17 min, Table 3).

**Table 3:** Pharmacokinetic parameters after single subcutaneous bolus administration of SNF472 into rats.

	10 mg/kg	30 mg/kg	100 mg/kg
**C**_**max**_ **(ng/mL)**	4876	61014	263264
**t**_**1/2**_ **(min)**	3.9	17.0	11.5
**AUC**_**(0→t)**_ **(ng·h/mL)**	1633	32646	139603
**MRT**_**(0→∞)**_ **(h)**	0.47	0.48	0.49
**Vz/F (L/kg)**	0.48	0.48	0.61
**Cl/F (L/h/kg)**	0.22	0.27	0.21

C_max_: maximum concentration measured; t_1/2_: half-life; AUC: area under the curve; MRT: mean residence time; Vz: volume of distribution; Cl: clearance; F: absolute bioavailability

The pharmacokinetic profiles of control and uremic rats receiving 10 and 50 mg/kg SNF472 via 4 h i.v. infusion are shown in Fig 2e and 2f, respectively. At a dose of 10 mg/kg SNF472, steady state was not achieved in control animals during the infusion period, reaching a maximum of 5548 ng/mL at 240 minutes of infusion. In contrast, maximum levels were reached within 30 min in uremic animals and low-level steady state observed at 60 min, with 1877 ng/mL SNF472 at 240 minutes (Fig 2g, Table 4). At a dose of 50 mg/kg, steady state was reached in control animals at 60 min (C_max_ of 45141 ng/mL) and between 10 and 30 min in uremic rats (C_max_ 16218 ng/mL) (Fig 2g, Table 4).

**Table 4:** Pharmacokinetic parameters after intravenous infusion administration of SNF472 into control and uremic rats.

	Day 1 (control)		Day 13 (uremic)	
	10 mg/kg	50 mg/kg	10 mg/kg	50 mg/kg
**C**_**max**_ **(ng/mL)**	5548	45141	1877	16218
**AUC**_**(0→t)**_ **(ng·h/mL)**	16027	145212	3877	51665

C_max_: maximum concentration measured. AUC: area under the curve. Uremia was induced by daily oral administration of 600 mg/kg adenine for 10 days and two oral administration of 300 ng/kg α-calcidol on days 11 and 13 to accelerate calcification.

### Inhibition of cardiovascular calcification by SNF472

#### Prevention of calcification induced by vitamin D

**Fig 3:**
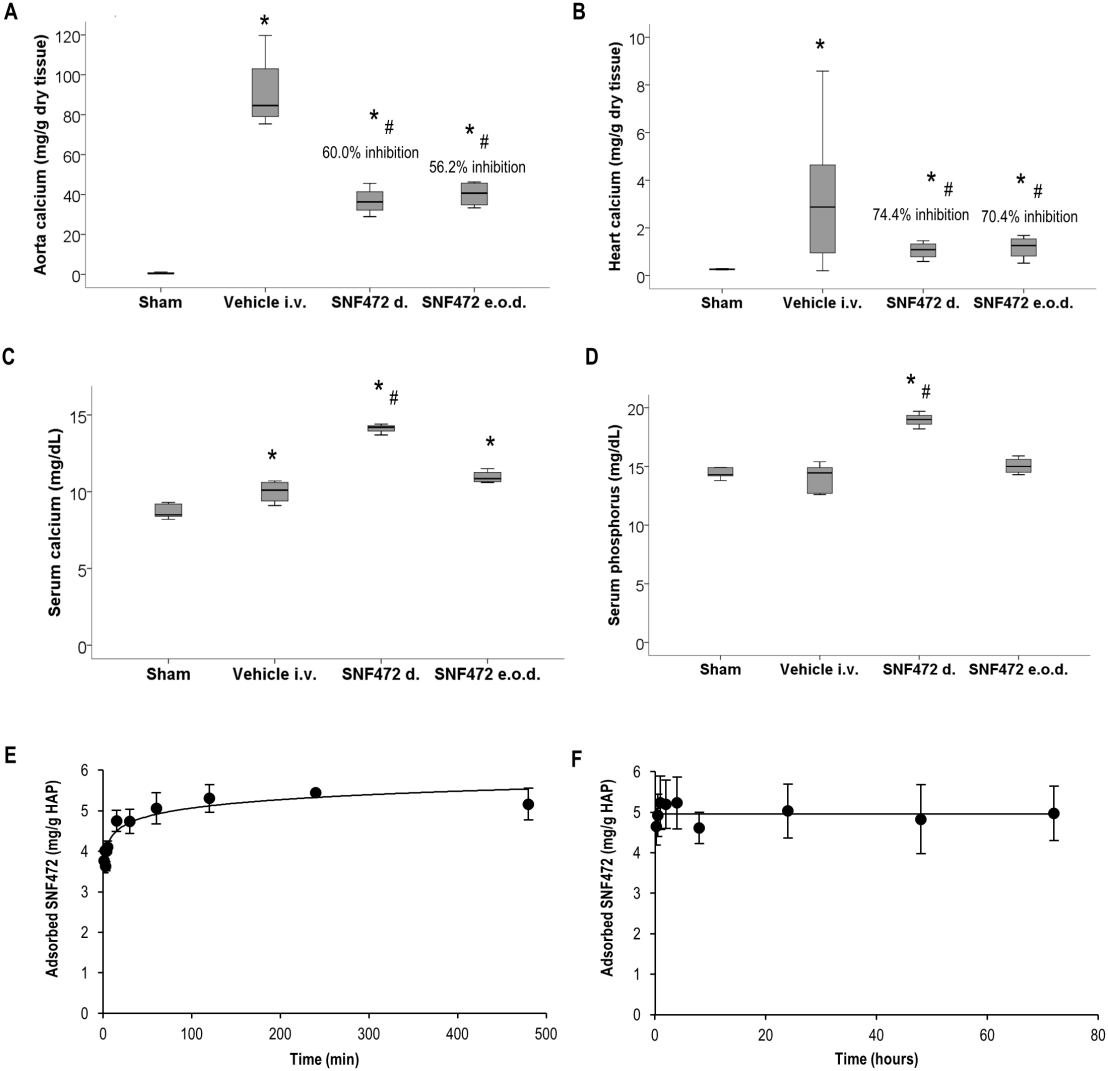
Preventive effects of SNF472 on cardiovascular calcification induced by vitamin D in rats. Effect on serum calcium and phosphorus levels in rats and *in vitro* binding studies. Calcium levels in aorta (A) and heart (B) and calcium (C) and phosphorus (D) levels in serum after calcification induction by 5 consecutive daily oral administrations of 75 kIU/kg vitamin D_3_ in control and treated animals. SNF472 d.: 1 mg/kg daily i.v. administration; SNF472 e.o.d.: 1 mg/kg every other day i.v. administration. (E) Binding kinetics of SNF472 on hydroxyapatite (HAP) assessed after incubation with 130 mg HAP in the presence of 5000 ng/ml SNF472 at 37 °C, pH 7.4. (F) Release kinetics of SNF472 from HAP. A solution of 5000 ng/ml SNF472 was incubated for 2 h at 37 °C in the presence of 130 mg HAP. HAP was recovered by filtration and incubated for up to 7 days in fresh 0.05 M Tris buffer, pH 7.40. (A, B, C, D) Boxplot representation of the results showing the median (horizontal black bar), Q1 (lower box), Q3 (upper box) and lower and upper limit. (E, F) Results represent mean ± SD. Statistical analysis: One-way ANOVA. (*) indicates significant differences vs. Sham group; (#) indicates significant differences vs. corresponding vehicle, p < 0.05. In vivo study (N = 5). In vitro study (N = 3).

Vitamin D_3_ administration for 5 days induced calcification in the aorta and heart (Fig 3). The calcium content was almost undetectable in sham rats but increased to mean values of 91.1 mg/g in the aorta (Fig 3a) and 3.35 mg/g in the heart (Fig 3b) after vitamin D_3_ treatment. Rats treated with SNF472 1 mg/kg daily developed aortic and heart calcium content of mean 36.8 and 1.06 mg/g respectively, which was significantly lower compared to the vehicle-treated group (mean 60 and 74% lower, respectively). Daily i.v. administration of SNF472 at a dose of 1 mg/kg resulted in mean aortic and heart calcium content of 40.2 and 1.18 mg/g respectively (60% and 74% statistically significant reduction in mean aortic and heart tissue calcification, respectively) (Fig 3a and 3b). Similar significant reductions were observed when SNF472 was administered e.o.d (mean inhibition of 56.2% and 70.4% in aorta and heart, respectively).

Serum calcium was significantly increased in vitamin D-treated animals (10.50 mg/dL), compared to sham animals (8.72 mg/dL, Fig 3c). The animals treated daily with i.v. SNF472 presented statistically significant higher levels than sham and control groups. No significant effects of vitamin D3 were evidenced in serum phosphorus levels. However, daily treatment with SNF472 resulted in significantly increased phosphorus levels in serum (Fig 3d) when compared to control animals (14.75 and 18.96 mg/dL in control and SNF472-treated animals, respectively). Fig 3E shows the *in vitro* binding and release kinetics of SNF472 on/from HAP. SNF472 bound to HAP crystals almost immediately, reaching 80% maximum adsorption after 5 min of incubation (Fig 3e). Maximum adsorption (~5 mg/g) was achieved at 60 min and plateaued after this time. The initial amount of SNF472 that adsorbed to HAP crystals remained stable after 3 days of incubation with fresh non SNF472-containing buffer (Fig 3f), confirming the high affinity of SNF472 for HAP.

#### Inhibition of calcification progression induced by vitamin D

**Fig 4:**
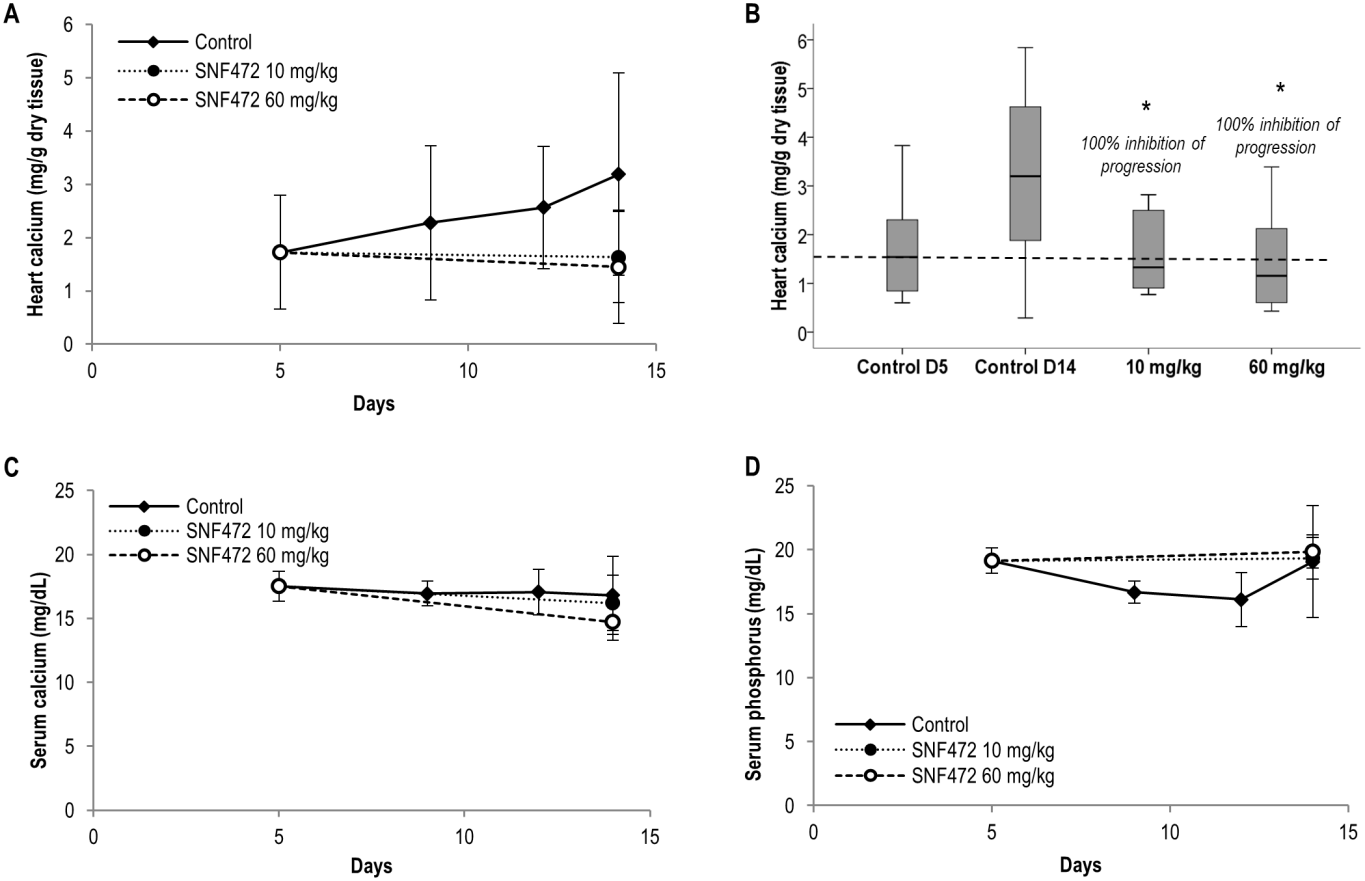
Effects of SNF472 on progression of vitamin D-induced cardiovascular calcification and serum and phosphorus levels in rats. (A) Heart calcification progression and (B) inhibition by SNF472 after calcification induction via 3 consecutive daily subcutaneous administrations of 100 kIU/kg vitamin D_3_. The dashed line indicates mean heart calcium levels by day 5 immediately before starting treatment with SN472. (C) Calcium and (D) phosphorus levels in serum of control and SNF472-treated animals. (A, C, D) Results represent mean ± SD. (B) Boxplot representation of the results showing the median (horizontal black bar), Q1 (lower box), Q3 (upper box) and lower and upper limit. Statistical analysis: One-way ANOVA. (*) indicates significant differences vs. control group, p < 0.05 (N = 8 per group).

Calcification in the heart progressed between days 5 and 14 after vitamin D administration (Fig 4a). Treatment with 10 and 60 mg/kg SNF472 administered s.c. completely inhibited further progression of cardiac calcification (Fig 4b). Serum calcium levels did not change in control animals between days 5 and 14 and were not significantly affected by SNF472 (Fig 4c). No differences in phosphorus levels were detected on day 14 between animals treated with SNF472 and their control counterparts (Fig 4d).

#### Inhibition of calcification in the rat model of uremia

**Fig 5:**
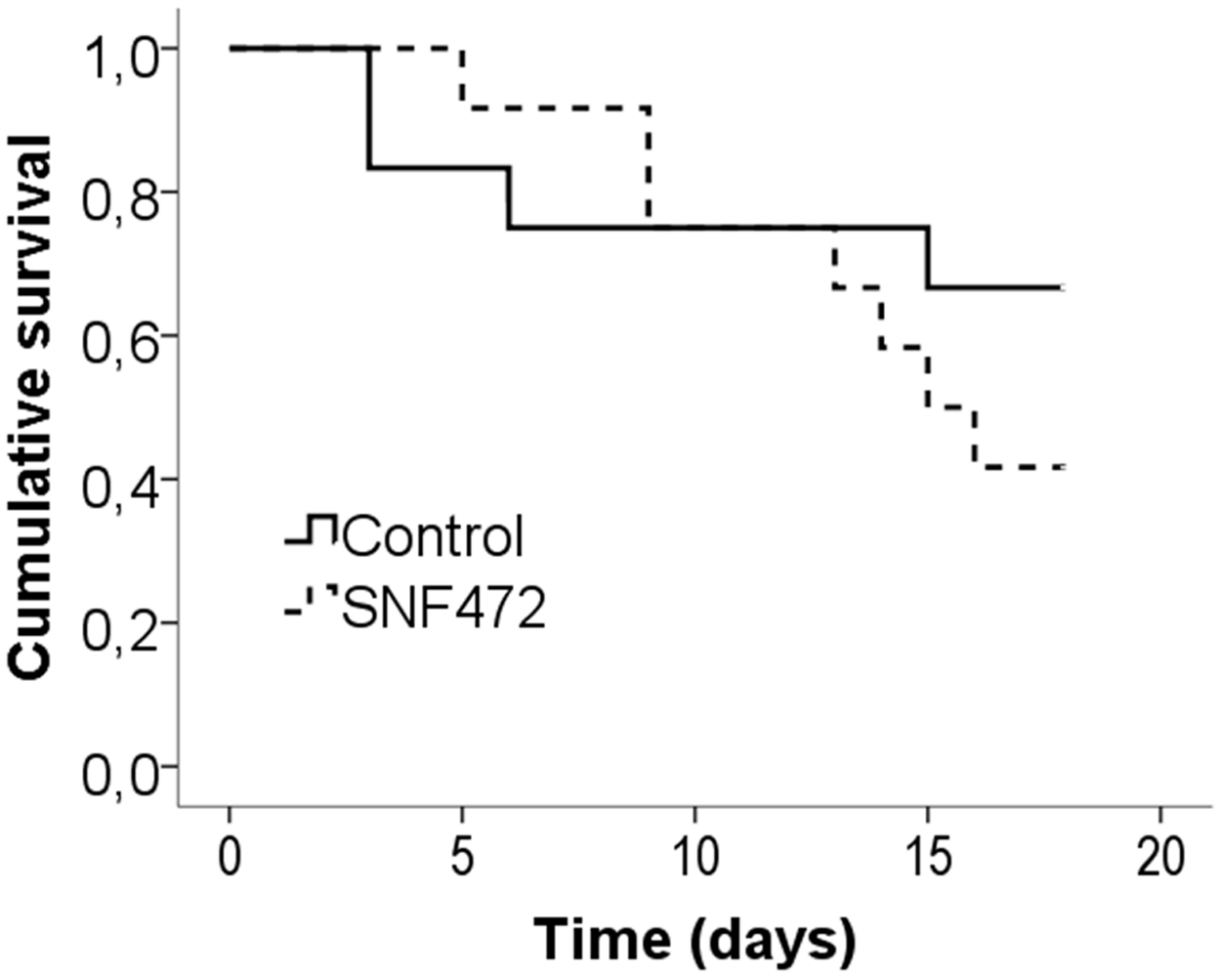
Survival in adenine-induced uremic rats.

Severe uremia resulted in high mortality (4 animals died in the control group and 7 animals died in the SNF472 group), but no significant differences were observed in mortality between the experimental groups (Fig 5). Therefore, the experiment was terminated on day 19, two days earlier than originally scheduled. Due to the low number of animals surviving on day 19 and considering the significant calcification on day 14, all animals that died between days 14 and 19 were included in the final analysis, with a final N of 9 rats in the control group and 8 rats in the SNF472-treated group.

**Fig 6:**
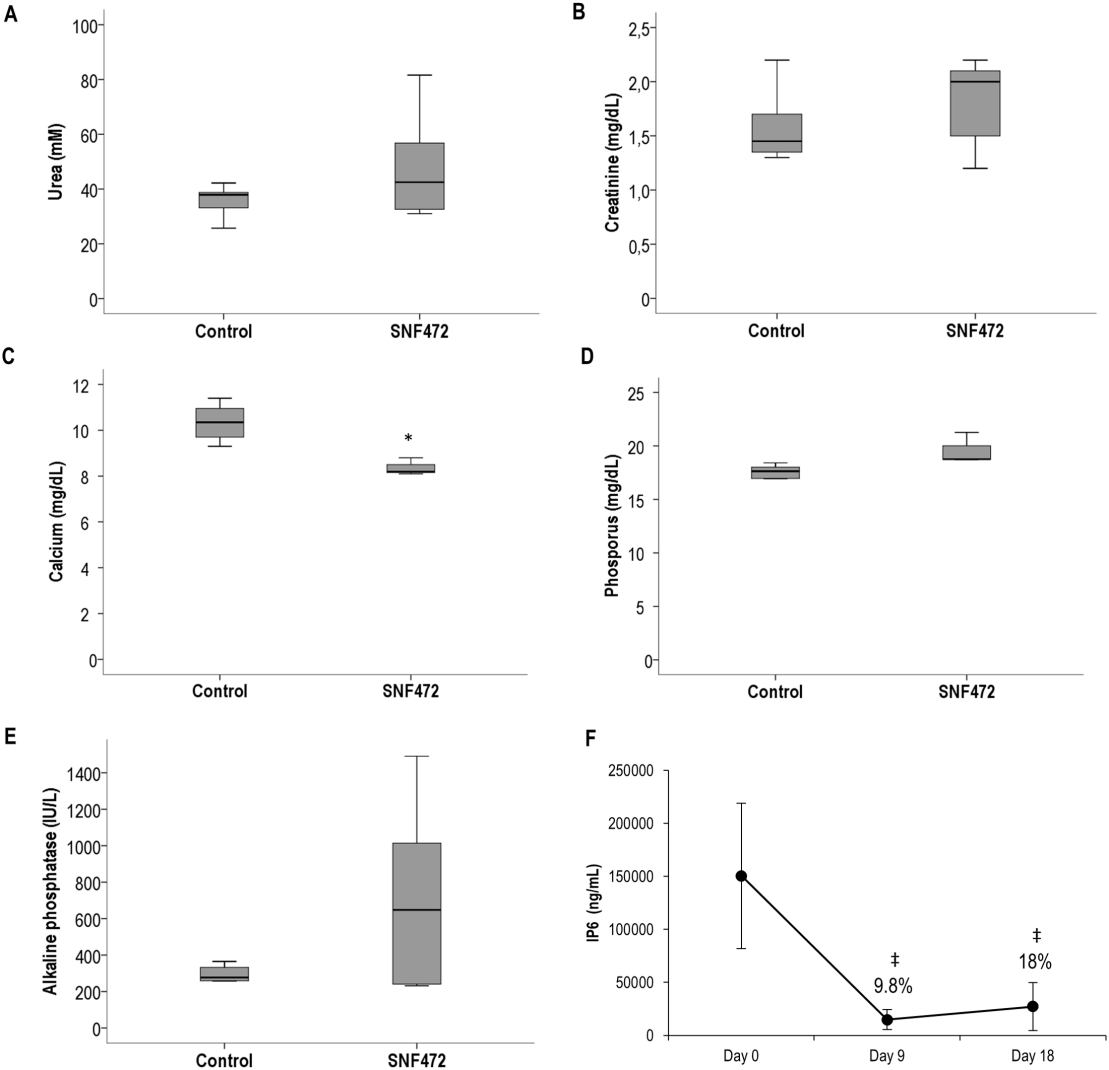
Biochemical parameters and SNF472 PK levels in adenine-induced uremic rats. (A) Urea, (B) creatinine, (C) calcium and (D) phosphorus levels and (E) alkaline phosphatase activity in serum of adenine-induced uremic rats treated with vehicle or SNF472 via 4 h intravenous infusion. (F) Phytate levels at the end of infusion (4 h) on days 0, 9 and 18 in the SNF472-treated group (phytate levels in control animals lower than the lower limit of quantification). (A, B, C, D, E) Boxplot representation of the results showing the median (horizontal black bar), Q1 (lower box), Q3 (upper box) and lower and upper limit. (F) Results represent mean ± SD. Statistical analysis: (A, B, C, D, E) Student’s t-test for independent samples, (F) one-way ANOVA. (*) indicates significant differences vs. control group; (‡) indicates significant differences vs. day 0, p < 0.05. Control group: N = 9; SNF472-treated group: N = 8.

No significant differences between control and SNF472-treated animals were observed regarding uremic parameters such as urea (Fig 6a) and creatinine (Fig 6b). A significant reduction (17%) in serum calcium levels was observed in animals treated with SNF472, but this was not accompanied by significant changes in circulating phosphorus (17.6 mg/dL in control group, 19.9 mg/dL in SNF472 group) (Fig 6c and 6d). Mean serum ALP activity was higher in SNF472-treated animals (725 U/L vs 295 U/L in controls) but this difference was not significant (Fig 6e).

**Fig 7:**
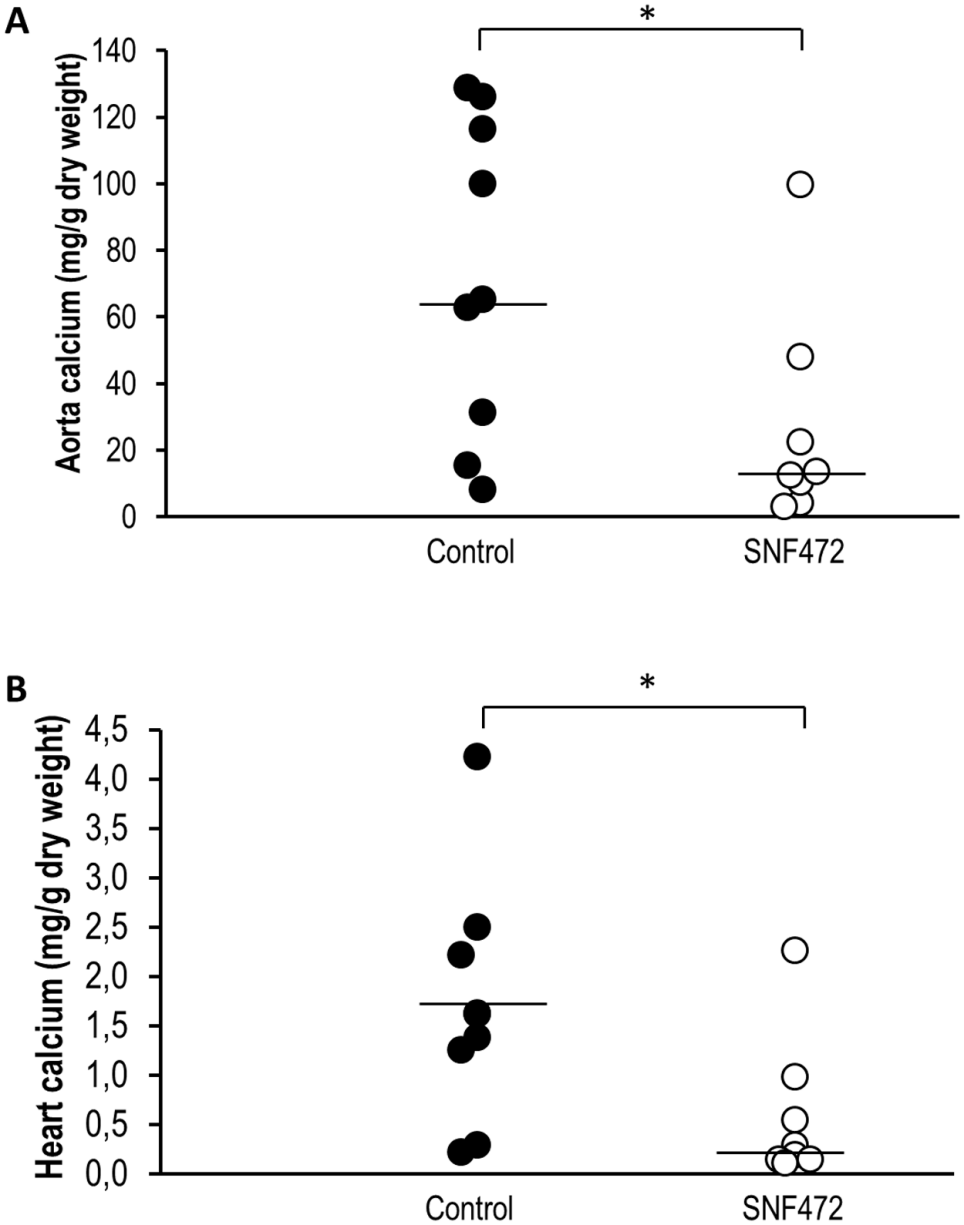
Inhibition of cardiovascular calcification in adenine-induced uremic rats via intravenous infusion of SNF472. (A) Aorta and (B) heart calcium contents in adenine-induced uremic rats treated with vehicle or SNF472 via 4 h intravenous infusion. Horizontal bars represent median values. Statistical analysis using Mann-Whitney U test. (*) indicates significant differences vs. control group, p < 0.05. Control group: N = 9; SNF472-treated group: N = 8.

The circulating phytate levels were ~150,000 ng/mL in non-uremic animals (Fig 5f). Uremic animals showed a 90% decrease in circulating phytate during and after 4 h of SNF472 infusion. The median aorta calcium content in SNF472-treated rats was significantly lower than that in control rats (Fig 7a, medians of 65.0 and 12.8 mg/g in control and SNF472-treated rats). Statistically significant lower levels of calcium in heart were also observed (Fig 7b, medians of 1.6 and 0.2 mg/g in control and SNF472-treated rats).

## Discussion

The pharmacological development of SNF472 as a treatment for vascular calcification presents a novel approach in the management of soft tissue calcification-based diseases, such as arteriosclerosis, atherosclerosis, cardiac valve calcification or calciphylaxis. While prevention of CVC by orally and subcutaneously administered SNF472 has been confirmed in rat models in which calcification is induced via other routines of vitamin D administration [23–25], to our knowledge, this is the first report on inhibition of progression of calcification and in a uremic rat model. At this stage, SNF472 is primarily being developed for use in ESRD patients on HD. In addition to hypercalcemia, which can result from vitamin D administration, renal failure is a key contributor to the development of vascular calcification in these patients. Pre-clinical studies on animal models with vitamin D- and uremia-induced calcification are therefore essential to develop appropriate treatment strategies.

In terms of pharmacokinetics in rats, SNF472 showed a low volume of distribution (0.05 L/kg) and clearance value of 0.26 L/h/kg after i.v. administration. The post-C_max_ half-life (T_1/2_) was short (<8 min), suggesting T_1/2α_ with a volume of distribution equal to the plasma volume. Following i.v. infusion, steady state was not achieved after 4 h, suggestive of at least one more compartment with T_1/2β_ >1 h. The pharmacokinetic profiles of SNF472 after i.v. and s.c. administration and i.v. infusion showed AUC dose proportionality. In uremic rats (induced by adenine), SNF472 plasma levels (C_max_ and AUC) were 3 to 4-fold lower than those in control animals, which might be attributable to hyperphosphatemia and upregulated ALP activity in untreated uremia. It has been previously reported that uremic rats present elevated activity of ALP, the enzyme that catalyzes the hydrolysis of pyrophosphate (PPi) to inorganic phosphate [30]. As other inositol phosphates, phytate is metabolized by subsequent hydrolysis of its phosphate groups. Although several intracellular inositol phosphatases have been described, no inositol phosphatase activities have been reported in circulation. At the view of our results, it would be interesting to ascertain whether phytate might be a substrate for ALP and whether increased ALP levels have an impact on SNF472 plasma levels. This is unlikely to be a major issue in ESRD patients on HD, as hyperphosphatemia is at least controlled to a better extent by HD and phosphate binders, compared to that in animal models with no intervention. Independently of the mechanism underlying SNF472 metabolism, the results obtained in this study evidence that the dosage of the compound has to be adjusted in uremic rats to compensate for the faster metabolism and achieve target exposure as well as inhibit calcification to the same extent as in non-uremic animals.

Data from the present study confirmed that SNF472 prevents the development of aortic and heart calcification induced by vitamin D in rats. Recent publications have confirmed inhibition of calcification by phytate administered subcutaneously [24], topically [23] and orally [26]. The mechanism by which SNF472 inhibits calcification differs from the indirect mechanisms underlying the suppressive effects of other compounds commonly used as therapy for advanced CKD (calcimimetics, phosphate binders or sodium thiosulfate). SNF472 exerts a physicochemical effect on HAP crystal formation, the final common pathway in the development of CVC, and is therefore expected to display inhibitory activity irrespective of etiology. Therapeutic levels of SNF472 inhibit the growth of HAP crystals at substoichiometric concentrations relative to free calcium (SNF472 concentrations in the order of micromolar inhibit the formation of hydroxyapatite when calcium is present at concentrations in the order of millimolar) without decreasing the circulating calcium levels. No decrease in the serum calcium level was observed in rats treated with i.v. or s.c. SNF472 bolus. Conversely, rats administered 1 mg/kg i.v. SNF472 daily had higher circulating levels of calcium than their control counterparts despite the lower degree of calcification, thus evidencing that the anti-crystallization properties of SNF472 is not related to calcium chelation The presence of high circulating SNF472 levels appears to inhibit the deposition of new calcium and phosphorus ions on growing HAP crystals, consequently delaying crystallization, even under conditions of elevated circulating calcium levels. At this point, it is difficult to explain why circulating calcium levels are increased after daily i.v. administration of SNF472, but as discussed in subsequent paragraphs, this phenomenon does not appear to be related to bone demineralization and loss of bone mass, but might be related to the model of calcification induction by vitamin D. As observed in the vehicle control group, the overdose of vitamin D induces an increase in circulating calcium, generating a situation of supersaturation that leads to the formation and deposition of calcium phosphate crystals in the blood vessels. When the animals are treated with SNF472, this acts as an inhibitor of calcium phosphate crystallization, and the non-crystallized calcium might remain in circulation, at least for the 14 days of study. When SNF472 was administered e.o.d., similar inhibition of CVC was observed. This finding may be explained by the high affinity of SNF472 for its target. As shown *in vitro*, once SNF472 attaches to HAP, binding persists for a relatively long period, allowing maintenance of efficacy when doses are administered e.o.d.

Both the degree and progression rate of CVC are related to lower survival and higher incidence of cardiovascular events [31–33]. Therefore, a potential drug targeting calcification should not only prevent but also slow down progression of calcification. Daily s.c. administration of >10 mg/kg SNF472 fully inhibited progression of heart calcification. Although we did not detect regression of calcification associated with SNF472 treatment in our experimental settings, full inhibition of calcification progression is of great importance. Regression of calcification is a slow process that requires more than just 9 days of study [34]. Therefore, longer progression studies should be performed to establish whether SNF472 can enhance calcium re-dissolution.

Although our results are promising, the mechanisms underlying calcification induced by high doses of vitamin D are not necessarily the same as those related to CVC associated with uremia. Differences in the nature of the deposited mineral material in calcified tissues have been described by Verberckmoes et al[35]: While apatite is the main mineral component in uremic rats with calcification enhanced by vitamin D, crystals of whitlockite have additionally been detected. Accordingly, we tested the effects of SNF472 on calcification induced in uremic rats. All animals in the study developed severe uremia, as evident from the high plasma levels of urea and creatinine, compared to non-uremic rats [5, 6]. Development of calcification was clearly inhibited by the compound, even with lower bioavailability, up to 80% and 85% in aorta and heart, respectively. A major concern was the elevated mortality rate in both experimental groups. The adenine dose was selected based on the results of Terai et al. [27] who reported no mortality with 600 mg/kg adenine and 50% mortality with 700 mg/kg (similar to that observed by our group treated with 600 mg/kg). In view of the unexpected high mortality, we performed a preliminary set-up study to determine whether a new experiment with a lower adenine dose should be carried out. Creatinine levels were significantly lower with 400 and 500 mg/kg than 600 mg/kg adenine (data not shown), highlighting a significantly lower degree of uremia. The differences in mortality between our study and that of Terai could be multi-factorial, but the major cause underlying high mortality is probably the catheterization of animals for i.v. infusion. This type of surgical intervention and maintenance of catheterization during the 3–4 weeks of study elevated stress in the animals, leading to the additional complication of negative effects on mortality. As SNF472 is under development for administration via i.v. infusion during dialysis, pharmacokinetics, pharmacodynamics and efficacy of the compound through this route are critical for clinical development.

A significant decrease in circulating calcium levels in uremic rats treated with SNF472 was observed. Calcium levels were assessed in serum samples without EDTA. In the absence of strong chelators, SNF472 at high concentrations binds to divalent cations, such as calcium, forming colloidal aggregates that are precipitated during blood centrifugation for serum collection. In addition, serum samples were diluted and filtered before analysis with the ICP-OES system. This process leads to elimination of potential SNF472-Ca aggregates. Therefore, a decrease in calcium at the end of SNF472 infusion (measured using this methodology) can be linked to chelation, which was observed specifically in the adenine study with high Cmax and AUC for SNF472. Therefore, this high exposure to SNF472 obtained with the 4-hour intravenous infusion may induce a slight calcium chelation. However, it must be noted that no signs of hypocalcemia (dyspnea, muscle spasms, convulsions) were reported at this dose. In relation to the chelating capacity of SNF472, an anti-tumor effect of phytate had previously been claimed based on its antioxidant capacity through iron chelation [36–38].

Other crystallization inhibitors with similar mechanisms of action as SNF472, such as pyrophosphate and bisphosphonates, have been examined in uremic rat models. Pyrophosphate was shown to inhibit uremic calcification induced by adenine and calcidol without exerting adverse effects on bone [39]. Bisphosphonates have anti-crystallization properties, but the doses required to inhibit CVC in uremic rats also suppress bone metabolism [40]. The finding that bisphosphonates are cleared by the kidneys and therefore accumulate in CKD and ESRD is an additional concern [41]. Our results are encouraging, since inhibition of the pathological process of calcification in soft tissue by SNF472 is associated with positive effects on bone. In an earlier report, ovariectomized Wistar rats fed a phytate-enriched diet showed reduced loss of bone mineral density caused by estrogen deficiency [20]. Two epidemiological studies on 1473 and 180 subjects revealed a correlation between higher bone mineral density and phytate consumption and physiological phytate levels, respectively [21, 22]. Another study on post-menopausal women demonstrated a correlation between high physiological levels of phytate and lower bone mass loss over a 12-month period [42]. Although phytate has been shown to inhibit mineralization of MC3T3–E1 osteoblast cultures *in vitro*,[43] incubation of human mesenchymal stem cells (pre-osteoblasts) with phytate in the micromolar range enhanced bone formation activity [44]. The observed effects of SNF472 on bone parameters and results obtained from 28-day and 39-week toxicology studies performed on rats and dogs (results presented as an oral communication [45]) suggest that undesired side-effects on bone are unlikely to occur. However, additional studies are required to discount the possibility of deleterious effects on bone, especially under uremic conditions. The study of bone properties in uremic animals is a compromised field due to the lack of true models that mimic the situation in humans. Recently, an alternative uremic rat model based on low-dose adenine administration was reported [46] and its suitability for testing the effects of SNF472 on bone should be considered in future studies.

In conclusion, SNF472 can reach potentially therapeutic levels by intravenous and subcutaneous administration. SNF472 presents a short half-life and bioavailability is reduced in uremic rats, indicating that higher doses must be administered in these animals when compared to non-uremic animals. SNF472 inhibits CVC in the three rat models by targeting the formation of HAP. In a uremic model involving daily infusion of 50 mg/kg SNF472 for 4 h, development of CVC was inhibited by up to 87.5% of calcium content. Similar degrees of inhibition were observed when calcification was induced in non-uremic animals by high doses of vitamin D, even when the calcification process had already started. SNF472 may therefore be considered a promising novel agent that effectively prevents progression of CVC. Moreover, in contrast to currently used compounds, such as phosphate binders, its physicochemical and therapeutic effects are independent of the etiology of CVC, as phytate specifically acts on the final common pathway of CVC; i.e. formation and growth of HAP crystals. We are currently in the process of completing a Phase 2 trial in ESRD patients. The data reported in this study support continued non-clinical research to obtain further insights into the mechanisms of action and effects of SNF472 on bone in addition to clinical evaluation through studies on patients on HD.
